# Association Between Smoking And Cancers Among Women: Results From The FRiCaM Multisite Cohort Study

**DOI:** 10.7150/jca.54624

**Published:** 2021-03-31

**Authors:** Angelo Giosuè Mezzoiuso, Anna Odone, Carlo Signorelli, Antonio Giampiero Russo

**Affiliations:** 1 Epidemiology Unit, Agency for Health Protection of Milan, Corso Italia 52, 20122, Milan, Italy.; 2 Faculty of Medicine, University Vita-Salute San Raffaele, Milan, Italy.; 3 Department of Medicine and Surgery, University of Parma, Parma, Italy.

**Keywords:** neoplasms, tobacco smoking, smoking, cohort studies, women's health.

## Abstract

**Background:** Smoking is one of the leading causes of death worldwide, and it is strongly associated with several human cancers. However, the differential effects of cigarette smoke on the development and progression of different types of cancer remain unclear, and related data are limited.

**Methods:** In this longitudinal cohort study conducted among 75,324 women aged 41-76 years, we aimed to evaluate the effect of exposure to tobacco smoke on cancer development. The participants completed a questionnaire assessing socio-demographic characteristics, anthropometric measures, health status, and lifestyle habits, including smoking and dietary habits; Cox proportional hazards regression modelling was used to evaluate the association between smoking and 21 different types of cancer.

**Results:** After a 15-year follow-up, we identified 9,487 cases of cancer through record linkage with the Cancer Registry of Milan. Smoking was found to be positively associated with all neoplasms, with a Hazard Ratio (HR) of 1.10 (95% Confidence Interval (CI), 1.04-1.16). Regarding the specific types, we found the following associations: cancer of the oral cavity HR = 2.63 ( 95% CI 1.72-4.01]), oesophagus HR = 3.09 (95% CI 1.37-6.96), stomach HR = 1.52 (95% CI 1.10-2.11), pancreas HR = 1.69 (95% CI 1.29-2.21), larynx HR= 34.81 (95% CI 8.07-150.14), lung HR = 8.48 (95% CI 7.09-10.14), cervix uteri HR = 2.51 (95% CI 1.38-4.57), and bladder and urinary tract HR = 5.67 ( 95% CI 3.96-8.14); lymphoma HR = 1.37 (95% CI 1.03-1.83); and colorectal cancer HR = 1.30 (95% CI 1.11-1.51).

**Conclusions:** Our results thus demonstrate how smoke exposure increases the risk of several types of cancer. Considering the increasing prevalence of smoking among women, our results highlight the need to prioritize the development of anti-smoking campaigns targeted at women in order to contrast the evident gender inequality with respect to healthcare.

## Background

Despite the downward trend in the incidence of cancer over the last few years, cancer remains the second leading cause of death after cardiovascular diseases in the states of the European Union. Cancer accounted for 26% of all deaths in 2013 1. Tobacco smoke has been recognised as an important risk factor for various human cancers and other chronic diseases, in female population, for several decades 2-4. For this reason, it is considered one of the largest threats to public health worldwide. According to the World Health Organization (WHO), tobacco causes more than seven million deaths each year, of which roughly six million are due to the direct use of tobacco and just below one million is associated with exposure to second-hand smoke 5.

According to the last report of the International Agency for Research on Cancer (IARC) of 2009, a cubic centimetre of smoke has approximately 4 × 10^9^ particles and over 5300 compounds, including monocyclic and polycyclic aromatic hydrocarbons, nitro compounds, and metals 4, 6. There is enough evidence demonstrating the carcinogenicity of at least 70 of these compounds 6, 7. However, this is not a current evaluation and it is probably underestimated. Indeed, these data are referred to the IARC report of 2009 and therefore they are antecedent to this period.

The latest WHO report (July 2019) on the global tobacco epidemic reported that there were an estimated 1.1 billion smokers in the world, and approximately 80% of them lived in low- and middle-income countries 8, 9. In the Italian population, the prevalence of smoking among those aged greater than 14 years was 19.7% in 2017; the prevalence was 24.8% among men and 14.9% among women, showing a strong gender difference 10. Interestingly, however, although the prevalence of smoking has been decreasing both among men and women for several decades, the mortality for cancers smoke-related is rising in women 11.

Substantial evidence on the relationship between smoking and several cancers has been obtained in the last few decades. Richard Doll and Bradford Hill conducted the first case-control study examining the association between smoking and cancer. They demonstrated a strong association between smoking and lung cancer, and tobacco smoke has now been recognised as the leading cause of lung cancer both among smokers and those exposed to second-hand smoke 3, 12-15.

However, tobacco smoke is also associated with several other types of cancer. Studies have examined the effect of tobacco smoke on the development of other types of cancer, such as cervical, bladder, and gastro-enteric cancer 16-25. Nevertheless, the evidence for the association between smoking and cancers other than lung cancer is limited and weak, particularly in female population.

Considering the evidence currently available with respect to the increase in the incidence of lung cancer in women, linked to the increase in exposure to cigarette smoke mainly in the female gender, it is essential to define the associations with other sites in order to identify the sites in which, in the coming years, an increase in cases is expected.

Therefore, the aim of this study was to evaluate the effect of exposure to tobacco smoke on the development of cancer at different sites using data from a large cohort of women in Italy and to further validate the current evidence on the relationship between smoking and lung cancer in this cohort.

## Methods

### Study cohort

For the present study, we used the prospective FRiCaM (Risk Factors for Breast Cancer, that is, “Fattori di Rischio per il Carcinoma della Mammella”) cohort.

Briefly, all women aged 41-76 years who resided in the municipality of Milan and were invited to undergo mammographic screening between 2003 and 2007 were included in the FRiCaM cohort. Details concerning the study design, recruitment, and questionnaire characteristics have been described previously 26.

Briefly, all participants completed a questionnaire on socio-demographic characteristics, anthropometric measures, health status, and lifestyle habits. In total, 131,246 women received a questionnaire and approximately 54% of them (71,398 women) completed it.

The questionnaire was also sent, by mail, to women who did not undergo mammographic screening in order to collect information from a sample of non-screened women, and approximately 33% of non-screened women (6,652 women) responded to the questionnaire.

Among the 78,050 women who answered the questionnaire, 2,726 were excluded owing to a lack of information on smoking habits. Thus, the final analysis in the present study was conducted among 75,324 women.

### Ethics

This is an observational study based on data routinely collected by the Agency for Health Protection (ATS) of Milan, a public body of the Regional Health Service - Lombardy Region, whose activity includes the evaluation of health status of the population. According to the regional law (R.L. 23/2015, 11/08/2015) http:/‌/norm‌elombardi‌a.consiglio‌.regi‌one.l‌om‌bardia.it‌/NormeLombardia/‌Acc‌essibile/main.aspx?view=showdoc&iddoc=‌lr002015081100023), ethical approval was deemed not necessary. This study is also ethically compliant with the National Law (D.Lgs. 101/2018 https://www‌.gazzettauffic‌‌iale.it/eli/id/2018/09/04/18G0012‌9/sg) and the “General Authorisation to Process Personal Data for Scientific Research Purposes” (n.8 and 9/2016, referred to in the Data Protection Authority action of 13/12/ 2018 https://www.garanteprivacy.it/home/docweb/-/docweb-display/docweb/9068972).

### Cancer types examined

During 15 years of follow-up, cases of cancer were identified through record linkage between the cohort study and the Cancer Registry of Milan. The Cancer Registry of Milan was accredited by IARC and has continuously collected all new invasive cancers from January 1999.

Starting from 2016, the Milan Municipality is part of cancer register of the Metropolitan area of Milan, included in Cancer Incidence in Five Continents - XI, covering the entire provinces of Milan for 3,176,180 inhabitants.

The different cancer types were coded using the 10^th^ revision of the International Classification of Diseases. The following 21 types of cancer were included: oral cavity (C00-C08), oesophagus (C09), stomach (C16), colon and rectum (C17-C21), liver (C22), gallbladder (C23-24), pancreas (C25), larynx (C32), bronchus and lung (C33-34), skin (including melanoma) (C43-44), breast (C50), cervix uteri (C53), corpus uteri (C54), ovary (C56), kidney (C64), bladder and urinary tract (C65-67), brain and nervous system (C71-72), and thyroid (C73) cancer; lymphoma (C81-88); multiple myeloma (C90); and all types of leukaemia (C91-95).

### Statistical analysis

Patients diagnosed with two or more primary cancers were included in the analyses for both cancer sites. Unconditional logistic regression analysis was used to assess the association between smoking habits disclosed in the questionnaire and several other covariates after adjustment for age to calculate the odds ratio (OR) and 95% confidence interval (CI). The chi-squared test was used to evaluate the differences in socio‐demographic characteristics, anthropometric measures, health status, and lifestyle habits between non-smokers and smokers stratified according to the number of cigarette pack-years.

Observation time was calculated from the date of enrolment until the date of diagnosis of each type of cancer included in the study, date of withdrawal from the study, date of death, or the end of the study period. We also estimated the smoking-related hazard ratios (HRs) and corresponding 95% CIs for each type of cancer using a Cox proportional hazard regression model adjusted for age and instruction level.

The correlation between smoking habits and cancer is proportional to the number of cigarettes smoked, which can be expressed in pack-years: the higher the number of cigarettes smoked, the higher is the risk of developing cancer. In this study, we considered cigarette pack-years as a categorical variable and stratified patients into four groups (less than 10, 10-20, 20-30, and more than 30 pack-years smoked) based on increasing exposure to smoke. We compared the risk of cancer development in smokers and ex-smokers with that in non-smokers, and further compared this risk between smokers stratified according to cigarette pack-years smoked and non-smokers. All analyses were performed using the SAS version 9.4 statistical software package (SAS institute Inc., Cary, NC, USA).

## Results

**Table 1** shows the detailed characteristics of the 75,324 participants included in the study, 16,144 (21.43%) of whom were smokers and 59,180 (78.57%) were never smokers or ex-smokers. According to our data, 56.4% of the smokers smoked at least 20 cigarettes/day and 32.3% smoked at least 30 cigarettes/day. Therefore, most smokers in the cohort were heavy smokers 27. Overall, the group exposed to tobacco smoke tended to be more educated, had a lower body mass index (BMI), and was younger than the non-exposed group.

The smokers in this study had a mean age of 58.2 years (standard deviation (SD) = 6.5 years) and a mean BMI of 23.5 kg/m^2^ (SD = 4.1 kg/m^2^). The non-smokers were older, with a mean age of 60.8 year (SD = 6.9 years), and their mean BMI was 24.6 kg/m^2^ (SD = 4.4 kg/m^2^). Moreover, 16.4% of the smokers went to university, 66.7% went to a secondary school, and 16.9% attended only primary school or did not attend any school at all. In contrast, only 15.1% of non-smokers completed university, 63.2% had a high school diploma, and 21.7% had completed only primary school.

With regard to marital status, 71.3% of non-smokers were married or cohabitant, whereas this rate was 63% among smokers; 10.1% of smokers and 8.1% of non-smokers were divorced. Moreover, 14.9% and 7.4% of smokers and non-smokers were widowed, respectively, and 12% and 13.2% were unmarried.

**Table 2** describes the relationship between dietary and smoking habits. In general, smokers had a lower intake of vegetables, fruit, fish, cheese, and white meat than non-smokers, and they consumed more red meat. Furthermore, the per-week portion consumption of fruits and vegetables appeared to decrease with an increase in the number of cigarettes smoked.

In addition, 75.7% of non-smokers consumed more than one portion of vegetables a day, while only 68.3% of smokers consumed the same amount of vegetables; this percentage decreased to 63.9% if only heavy smokers were considered. Similarly, 89.1% of non-smokers consumed one portion of fruit a day; this rate was 76.5% among smokers and 70% among heavy smokers. Only small differences in the consumption of other foods were noted between smokers and non-smokers (**Table 2**).

The risk estimates for the association between cigarette smoking and cancer are illustrated in **Table 3.** Cancer was found to have developed in 9,487 cases, representing 12.2% of the cohort. Overall, the person-time incidence for all cancers included was 112.09 person-years for every 10,000 subjects among smokers and 90.96 person-years for every 10,000 subjects among smokers and ex-smokers. A positive association was found between smoking and all types of cancers, with an HR = 1.10 (95% CI 1.04-1.16, Χ^2^ < 0001) for smokers and HR = 1.38 (95% CI 1.31-1.45) in ex-smokers. The risk increased with the number of cigarettes smoked, from 1.07 (95% CI 0.94-1.21) among those who smoked less than 10 pack-years to 1.63 (95% CI 1.51-1.76) among those who smoked more than 30 pack-years.

Smoke exposure was positively associated with several types of cancer, as follows: colorectal HR = 1.30 (95% CI 1.11-1.51, Χ^2^ = 0.0015) among smokers and HR = 1.16 (95% CI 1.00-1.35) among ex-smokers, laryngeal HR = 34.81 (95% CI 8.07-150.14, Χ^2^ = 0.0019) among smokers and 8.31 (95% CI 1.66-41.43) among ex-smokers, lung HR = 8.48 (95% CI 7.09-10.14, Χ^2^ < 0001), among smokers and 3.07 (95% CI 2.49-3.78) among ex-smokers, cervical HR = 2.51 (95% CI 1.38-4.57, Χ^2^ = 0.019) among smokers and HR = 1.91 ( 95% CI 1.02-3.59) among ex-smokers, and bladder cancer HR = 5.67 (95% CI 3.96-8.14, Χ^2^ < 0001) among smokers and HR = 2.37 (95% CI 1.56-3.60) among ex-smokers. A negative correlation was found for uterine and breast cancer, with an HR = 0.82 (95% CI 0.64-1.06, Χ^2^ = 0.015) for uterine cancer among smokers and an HR = 0.75 (95% CI 0.58-0.96) among ex-smokers, and an HR = 0.96 (95% CI 0.87-1.05, Χ^2^ = 0.036) for breast cancer among smokers and an HR = 0.91 (95% CI 0.83-0.99) among ex-smokers. For cancer of the oral cavity HR = 2.63 (95% CI 1.72-4.01), oesophagus HR = 3.09 (95% CI 1.37-6.96), stomach HR = 1.52 (95% CI 1.10-2.11), and pancreas HR = 1.69 (95% CI 1.29-2.21) and for lymphomas HR = 1.37 (95% CI 1.03-1.83), current smoking status was associated with cancer development, without any trend effect (**Table 2**).

The risk of cancer increased with the number of cigarettes. In more detail, for laryngeal cancer, the risk increased from HR = 12.37 (95% CI 2.22-68.92), for those who smoked less than 10 pack/year, to HR = 22.76 (95% CI 6.75-76.80) for heavy smokers. For lung cancer, from HR = 1.17 (95% CI 0.64-2.15) to HR = 12.03 (95% CI 9.99-14.49). For cervical cancer, from HR = 1.71 (95% CI 0.40-7.28) to 2.48 (95% CI 1.01-6.08), and for bladder cancer, from HR=2.53 (95% CI 1.01-6.35) to HR = 7.40 (95% CI 4.79-11.45). In contrast, colorectal cancer bucked the trend, for we observed a decrease with the number of cigarettes smoked, from HR = 1.56 (95% CI 1.12-2.18) for people that smoked less than 10 pack/years of cigarettes to HR = 1.13 (95% CI 0.86-1.47) for people that smoked more than 30 pack/year (**Table 2**).

## Discussion

In this study, which aimed to analyse the association between cigarette smoke and the development of different types of cancer in women, we evaluated a cohort of 75,324 female residents in the municipality of Milan who underwent mammographic screening and answered a questionnaire concerning socio-demographic data, anthropometric measures and information regarding their lifestyle, including food and smoke. Through linkage with the Cancer Registry of Milan, we found that 9,487 (12.6%) women from the cohort were diagnosed with cancer after enrolment.

Although just above half of the potential candidates filled out the questionnaire, this does not configure a selection bias, but a non-differential selection, which does not alter the results. In fact, the dependence of the outcome, the occurrence of cancer, is consistent across the exposure categories.

The present study showed that smoking was positively associated with an increased risk of several types of cancer, including tracheal, bronchial and lung, laryngeal, bladder and urinary tract, cervical, and colorectal cancer.

For colorectal cancer, the HR was 1.30 (95% CI 1.11-1.51) in the smoker population. This association is not well demonstrated in the literature. Studies conducted before 1980 failed to identify any association between cigarette smoking and colorectal cancer. Over the following twenty years, some studies found an association between long-term exposure to smoke and colorectal cancer among heavy smokers 28. Giovannucci et al, found an association between smoking and the presence of colorectal cancer in women (Relative Risk (RR) =1.11; 95% CI = 0.93-1.34) 35 years after smoking initiation 29. Similar results were noted in cohort and case-control studies 30-35. A more recent meta-analysis of observational studies conducted by Yang et al, in 2016 found an association between passive smoke exposure and rectal cancer RR=1.14 (95% CI 1.05-1.24) 36 Therefore our data are coherent with the data present in literature. However further studies are needed in order to investigate in more detail this association.

Interestingly, in this study we found a negative association between smoking and uterine cancer, with an HR of 0.82 (95% CI 0.64-1.06) thus not significant, but a significant HR of 0.75 (95% CI 0.58-0.96) in ex-smokers. These results are consistent with the current literature 37.

Breast cancer was negatively associated with cigarette smoking, with an HR of 0.96 (95% CI 0.87-1.05) for smokers and of 0.91 (95% CI 0.83-0.99) for ex-smokers. As previously, the results are significant only for ex-smokers.

Regarding this association, results in literature found an increased association both for breast cancer-specific mortality and for the all-cause mortality in smokers, while in ex-smokers, is mildly increased the all-cause mortality but not the breast cancer-specific mortality 38. Therefore, this association is controversial.

In our opinion it is unlikely that smoke have a real protective role. Our results and the results in literature could be explained by considering the composition of our cohort. Indeed, the smokers of the FRiCaM cohort study are mainly heavy smokers. It is possible that ex-smokers have a decreased risk of dying for breast and uterine cancer because they were exposed to high level of carcinogenic and so, they have a higher probability of dying for other smoke-related diseases such as cardiovascular diseases or lung cancer. Another explanation could be the presence of confounders or bias.

In a recent study, Pakzad et al, performed a case-control study and corrected the association between breast cancer and smoke for the smoking misclassification bias secondary to self-reporting. After this adjustment they found that the association between breast cancer and smoke, previously negative became positive 39. Therefore, further studies are needed in order to evaluate this association, and possible bias of misclassification should be considered.

Further, the present study also confirmed an association between smoking and cancer of the lung, bronchi, trachea, larynx, bladder, and pancreas. As previously written, the smokers in this cohort were largely heavy smokers, which clearly affected the results. Smokers had a higher risk of developing lung cancer (HR=8.48 vs. non-smokers). When we considered exposure-related stratification, we found that the risk among those smoking <10 pack-years versus the risk among never and ex-smokers combined was 1.17. In contrast, when the exposure was ≥30 pack-years, the risk was considerably higher at 12.03, clearly demonstrating that heavy smokers contributed highly to the first HR of 8.48. The HR of 1.17 noted for those consuming <10 pack/years can be explained by the fact that this risk was measured in relation to the risk among never and ex-smokers combined, and despite the reduction in the risk observed after quitting smoking, the risk among ex-smokers is still higher than that among never smokers.

This study has some limitations that must be considered, the most important of which was the low number of cases of some types of cancer, that made it difficult to look for associations with smoke. This limitation could be addressed, in future studies, by collecting more data and following the cohort for a more extended period.

Despite these, the study has several strengths: the cohort size, the long follow-up duration of 15 years, and the significant predominance of heavy smokers in the exposed group.

## Conclusions

In conclusion, the evidence from this population-based cohort study confirms that cigarette smoke increases the overall risk of cancer and specifically raises the risk of cancer of the lungs, bronchi, trachea, larynx, colon, pancreas, and cervix. One important point of our study is that we identified an increased risk of multiple types of cancer, for which there was previously little evidence. Secondly, the present study was conducted considering the increasing cancer incidence among women due to the rising trend of smoking habits in this group. This trend is expected to expand the current gender gap in healthcare. Our results thus highlight the need to develop effective anti-tobacco health initiatives targeting women, such as campaigns for smoking reduction, mass advertising campaigns in the media, and health education at school, which if conducted properly and for extended durations, could effectively reduce the current gender inequalities in healthcare.

## Figures and Tables

**Table 1:** Distribution of socio-demographic and individual characteristics among participants of the FRiCaM cohort study (ATS Milan 2019).

	Smokers	Non-smokers	OR	<10 pack/year	10-19 pack/year	20-29 pack/year	≥30 pack/year	Χ^2^
Age								
<50	1041 (6.5%)	2384 (4%)	1*	175 (8%)	335 (8.4%)	207 (6.1%)	204 (4.5%)	<0.0001
50-54	3496 (21.6%)	8444 (14.3%)	1.06	568 (26%)	1001 (25.3%)	701 (20.6%)	843 (18.6%)	
55-59	4304 (26.7%)	12362 (20.9%)	1.25	595 (27.2%)	1079 (27.2%)	868 (25.5%)	1205 (26.5%)	
60-64	3478 (21.5%)	12875 (21.8%)	1.62	402 (18.4%)	725 (18.3%)	797 (23.4%)	1116 (24.6%)	
65-69	2495 (15.5%)	13879 (23.4%)	2.43	318 (14.5%)	530 (13.4%)	522 (15.4%)	779 (17.1%)	
≥70	1330 (8.2%)	9236 (15.6%)	3.03	129 (5.9%)	293 (7.4%)	306 (9%)	395 (8.7%)	
Mean	58.2 (44-75)	60.8 (41-76)						
Total	16144	59180		2187 (15.5%)	3963 (28.1)	3401 (24.2%)	4542 (32.2%)	
Year of enrolment								<0.0001
2003	3696 (22.9 %)	13866 (23.4 %)	1*	524 (24%)	933 (23.5%)	800 (23.5%)	1014 (22.3%)	
2004	7225 (44.7 %)	27034 (45.7 %)	1.02	1007 (46%)	1732 (43.7%)	1478(43.5%)	2064 (45.4%)	
2005	4615 (28.7 %)	16469 (27.8 %)	1.02	570 (26%)	1118 (28.2%)	1007(29.6%)	1307(28.8%)	
2006	510 (3.1%)	1472 (2.5%)	1.01	68 (3.2%)	152 (3.9%)	97 (2.8%)	131 (2.9%)	
2007	98 (0.6%)	339 (0.6%)	1.06	18 (0.8%)	28 (0.7%)	19 (0.6%)	26 (0.6%)	
Education^1^								<0.0001
Primary school	2712 (16.9 %)	12721 (21.7 %)	1.04	311 (14.3%)	603 (15.3%)	569 (16.9%)	717 (15.9%)	
Secondary school	10685 (66.7 %)	37124 (63.2 %)	0.92	1361 (62.4%)	2631 (66.9%)	2265 (66.9%)	3123 (69.4%)	
University	2629 (16.4 %)	8870 (15.1 %)	1*	509 (23.3%)	702 (17.8%)	549 (16.2%)	663 (14.7%)	
Work Activity								
Manager/Professional/Teacher	3228 (20.7 %)	10941 (19.1 %)	1*	564 (26.5%)	849 (22%)	673 (20.4%)	878 (19.9%)	
Employee/merchant/artisan	6827 (43.7 %)	22370 (39 %)	0.90	903 (42.4%)	1673 (43.4%)	1444 (43.7%)	2024 (45.8%)	<.0001
Clerk/technician	670 (4.3 %)	2298 (4 %)	0.87	79 (3.7%)	200 (5.2%)	123 (3.7%)	178 (4%)	
Skilled worker	747 (4.8%)	2987 (5.2 %)	0.96	88 (4.1%)	171 (4.4%)	165 (5%)	202 (4.6%)	
Worker	1068 (6.8%)	4218 (7.3 %)	0.98	103 (4.8%)	242 (6.3%)	223 (6.7%)	285 (6.5%)	
Housewife	2979 (19.1%)	14178 (24.7 %)	1.13	380 (17.9%)	696 (18.1%)	655 (19.9%)	816 (18.5%)	
Never worked	99 (0.6%)	404 (0.7 %)	0.94	12 (0.6%)	22 (0.6%)	19 (0.6%)	33 (0.7%)	
Marital status^1^								<.0001
Married/cohabitant	9882 (63%)	41048 (71.3%)	1*	1427 (66.8%)	2600 (67.2%)	2110 (63.6%)	2558 (57.9%)	
Separated/divorced	1587 (10.1%)	4652 (8.1%)	0.70	192 (9%)	333 (8.6%)	356 (10.7%)	514 (11.6%)	
Widow	2344 (14.9%)	4230 (7.4%)	0.47	289 (13.5%)	522 (13.6%)	471 (14.2%)	774 (17.5%)	
Never married	1885 (12%)	7629 (13.2%)	0.73	229 (10.7%)	411 (10.6%)	383 (11.5%)	574 (13%)	
Age at menarche (years)^2^								<.0001
≤11	4258 (27 %)	13674 (23.7 %)	1*	528 (24.5%)	1049 (26.9%)	909 (27.4%)	1238 (28%)	
12-13	7728 (49 %)	28791 (49.8 %)	1.12	1135 (52.8%)	1911 (49%)	1627 (49%)	2133 (48.2%)	
≥14	3788 (24 %)	15288 (26.5%)	1.10	489 (22.7%)	941 (24.1%)	783 (23.6%)	1055 (23.8%)	
Age at first live birth (years)^2^								<.0001
Nulliparous	2924 (18.7%)	8762 (15.3%)						
≤20	1462 (9.4 %)	3632 (6.3%)	1*	140 (8.1%)	318 (10%)	282 (10.6%)	478 (13.8%)	
21-24	3674 (23.6%)	13394 23.3%)	1.36	445 (25.7%)	937 (29.5%)	737 (27.9%)	1018 (29.3%)	
25-29	4798 (30.8%)	20661 (36%)	1.51	736 (42.4%)	1205 (37.9%)	1080 (40.8%)	1246 (35.9%)	
≥30	2737 (17.5%)	10962 (19.1%)	1.46	413 (23.8%)	716 (22.6%)	547 (20.7%)	729 (21%)	
Menopausal status								<.0001
Pre-menopause	2771 (18.5 %)	8227 (15.2 %)	1*	516 (24.9%)	821 (22.2%)	565 (17.7%)	571 (13.4%)	
Post-menopause	12228(81.5 %)	45983 (84.8 %)	0.73	1559 (75.1%)	2883 (77.8%)	2633 (82.3%)	3688 (86.6%)	
BMI								
<18.5	977 (6.3%)	1936 (3.4%)	0.63	131 (6.2%)	2441 (63.8%)	200 (6.1%)	280 (6.4%)	<.0001
18.5-24.9	9521 (61.5%)	30372 (54%)	1*	1362 (64.5%)	242 (6.3%)	2063 (63.1%)	2535 (58.2%)	
25-29.9	3939 (25.5%)	18152 (32.3%)	1.36	494 (23.4%)	922 (24%)	816 (25%)	1165 (26.7%)	
≥30	1036 (6.7%)	5721 (10.2%)	1.65	125 (5.9%)	223 (5.8%)	189 (5.8%)	380 (8.7%)	
Mean	23.5 (DS=4.1)	24.6 (DS=4.4)						
Total	16144	59180						

**Table 2:** Distribution of dietary habits among participants of the FRiCaM cohort study (ATS Milan 2019).

One portion of vegetables	Smokers	Non-smokers	OR	<10 pack/year	10-19 pack/year	20-29 pack/year	≥30 pack/year	Χ^2^
**Less than once a week**	385 (2.4%)	797 (1.4%)	0.50	31 (1.4%)	60 (1.5%)	67 (2%)	165 (3.7%)	<.0001
**1 to 6 times a week**	4609 (29.3%)	13237 (22.9%)	0.71	520 (24.4%)	1022 (26.3%)	995 (29.7%)	1449 (32.4%)	
**Once a day or more**	10763(68.3%)	43801 (75.7%)	1*	1582 (74.2%)	2804 (72.2%)	2289 (68.3%)	2854 (63.9%)	
**One portion of fruit**								<.0001
**Less than once a week**	660 (4.2%)	661 (1.1%)	0.48	41 (1.9%)	112 (2.9%)	138 (4.2%)	296 (6.7%)	
**1 to 6 times a week**	3013 (19.3%)	5644 (9.8%)	0.73	348 (16.3%)	683 (17.7%)	615 (18.6%)	1023 (23.3%)	
**Once a day or more**	11925 (76.5%)	51287 (89.1%)	1*	1751 (81.8%)	3060 (79.4%)	2560 (77.2%)	3078 (70%)	
**One portion of cheese**								<.0001
**Less than once a week**	1294 (8.4%)	3868 (6.9%)	1*	146 (6.9%)	296 (7.8%)	253 (7.8%)	437 (10%)	
**1 to 6 times a week**	11003(71.7%)	39958 (70.8%)	1.31	1546 (73.6%)	2774 (73.2%)	2364 (72.7%)	3029 (69.5%)	
**Once a day or more**	3047 (19.9%)	12581 (22.3%)	1.96	410 (19.5%)	720 (19%)	635 (19.5%)	891 (20.5%)	
**One portion of red meat**								<.0001
**Less than once a week**	3823 (24.7%)	14490 (25.6%)	1*	492 (23.3%)	913 (23.8%)	813 (24.8%)	1165 (26.5%)	
**1 to 6 times a week**	11111 (71.9%)	40481 (71.4%)	1.12	1558 (73.8%)	2825 (73.7%)	2384 (72.7%)	3026 (69%)	
**Once a day or more**	532 (3.4%)	1705 (3%)	1.64	62 (2.9%)	95 (2.5%)	82 (2.5%)	196 (4.5%)	
**One portion of white meat**								<.0001
**Less than once a week**	2732 (17.7%)	7233 (12.7%)	0.52	314 (14.9%)	620 (16.2%)	547 (16.5%)	935 (21.4%)	
**1 to 6 times a week**	12236 (79.2%)	47686 (83.5%)	0.66	1729 (82.2%)	3099 (81.1%)	2664 (80.6%)	3285 (75.3%)	
**Once a day or more**	487 (3.1%)	2165 (3.8%)	1*	61 (2.9%)	103 (2.7%)	95 (2.9%)	140 (3.3%)	
**One portion of fish**								<.0001
**Less than once a week**	5330 (34%)	16282 (28.2%)	0.53	627 (29.3%)	1202 (31.1%)	1155 (34.6%)	1722 (38.8%)	
**1 to 6 times a week**	10195 (65%)	40747 (70.6%)	0.69	1492 (69.6%)	2633 (67.9%)	2157 (64.7%)	2674 (60.2%)	
**Once a day or more**	167 (1%)	691 (1.2%)	1*	23 (1.1%)	40 (1%)	23 (0.7%)	46 (1%)	

**Table 3:** Hazard Ratios and corresponding confidence intervals for smokers vs non-smokers (ATS Milan 2019).

	Incidence (*10000)			Smoker			Pack-Years				*Chi-square for trend*
	*Number of cases*	*Exp*	*Non Exp*	*No*	*Ex*	*Yes*	*<10*	*10-19*	*20-29*	*>30*	
Oral cavity	*112*	1.97	0.85	1*	*1,10* *(0.65-1.87)*	*2.63* *(1.72-4.01)*	*1.31* *(0.40-4.23)*	*2.21* *(1.07-4.54)*	*1.71* *(0.73-4.01)*	*4.88* *(2.94-8.11)*	0.19
Oesophagus	33	0.59	0.25	1*	1.41 (0.55-3.59)	3.09 (1.37-6.96)	0(0-)	1.13 (0.15-8.74)	3.53 (1.00-12.51)	5.90 (2.33-14.96)	0.26
Stomach	225	2.48	2.105	1*	1.03 (0.72-1.46)	1.52 (1.10-2.11)	0.84 (0.31-2.29)	1.17 (0.61-2.24)	1.39 (0.75-2.59)	2.08 (1.32-3.28)	0.46
Colon & Rectum	1080	11.03	10.34	1*	1.16 (1.00-1.35)	1.30 (1.11-1.51)	1.56 (1.12-2.18)	1.30 (0.99-1.71)	1.33 (1.00-1.76)	1.13 (0.86-1.47)	0.015
Liver	143	1.33	1.403	1*	0.84 (0.54-1.30)	1.15 (0.75-1.76)	n.a.**	1.14 (0.53-2.48)	1.60 (0.80-3.18)	0.80 (0.35-1.84)	0.76
Gallbladder	114	1.33	1.05	1*	1.17 (0.73-1.87)	1.54 (0.97-2.43)	1.19 (0.37-3.82)	1.78 (0.84-3.76)	0.99 (0.36-2.73)	2.05 (1.07-3.91)	0.33
Pancreas	309	3.77	2.79	1*	1.18 (0.89-1.58)	1.69 (1.29-2.21)	1.39 (0.71-2.73)	1.50 (0.92-2.46)	1.56 (0.94-2.58)	1.93 (1.29-2.89)	0.067
Larynx	29	0.965	0.098	1*	8.31 (1.66-41.43)	34.81 (8.07-150.14)	12.37 (2.22-68.92)	13.51 (3.31-55.12)	18.79 (4.98-70.98)	22.76 (6.75-76.80)	0.0019
Bronchus and lung	829	21.14	4.54	1*	3.07 (2.49-3.78)	8.48 (7.09-10.14)	1.17 (0.64-2.15)	3.73 (2.81-4.95)	5.90 (4.60-7.57)	12.03 (9.99-14.49)	<.0001
Skin cancers (including melanoma)	1429	11.48	14.51	1*	1.07 (0.94-1.22)	0.90 (0.78-1.04)	0.78 (0.53-1.13)	1.04 (0.81-1.33)	1.04 (0.80-1.35)	0.85 (0.66-1.09)	0.47
Breast	2952	28.21	28.77	1*	0.91 (0.83-0.99)	0.96 (0.87-1.05)	0.97 (0.98-0.78)	0.88 (0.74-1.05)	1.12 (0.95-1.33)	0.99 (0.85-1.16)	0.036
Cervix uteri	62	0.965	0.504	1*	1.91 (1.02-3.59)	2.51 (1.38-4.57)	1.71 (0.40-7.28)	2.76 (1.12-6.81)	2.70 (1.03-7.11)	2.48 (1.01-6.08)	0.019
Corpus uteri	428	3.77	4.26	1*	0.75 (0.58-0.96)	0.82 (0.64-1.06)	0.74 (0.39-1.40)	0.91 (0.58-1.40)	0.72 (0.43-1.21)	0.98 (0.66-1.46)	0.015
Ovary	273	2.297	2.74	1*	1.04 (0.78-1.34)	0.88 (0.64-1.21)	1.12 (0.58-2.19)	0.83 (0.46-1.49)	0.98 (0.54-1.75)	0.43 (0.20-0.92)	0.91
Kidney	167	1.56	1.64	1*	1.10 (0.75-1.60)	1.11 (0.74-1.65)	1.41 (0.62-3.25)	0.39 (0.12-1.24)	1.02 (0.47-2.12)	1.41 (0.79-2.52)	0.58
Bladder. urinary tract	170	3.63	1.12	1*	2.37 (1.56-3.60)	5.67 (3.96-8.14)	2.53 (1.01-6.35)	3.06 (1.59-5.87)	6.87 (4.21-11.21)	7.40 (4.79-11-45)	<.0001
Brain and nervous system	123	1.485	1.21	1*	1.44 (0.94-2.21)	1.18 (0.74-1.91)	1.12 (0.35-3.58)	1.26 (0.54-2.92)	1.84 (0.88-3.86)	0.69 (0.25-1.90)	0.09
Thyroid	159	1.56	1.54	1*	0.89 (0.60-1.32)	0.92 (0.61-1.37)	1.12 (0.48-2.56)	0.95 (0.47-1.89)	0.76 (0.33-1.73)	0.68 (0.32-1.47)	0.53
Lymphomas	298	3.26	2.79	1*	1.20 (0.90-1.59)	1.37 (1.03-1.83)	1.57 (0.85-2.90)	1.30 (0.77-2.18)	1.62 (0.99-2.65)	1.08 (0.64-1.81)	0.12
Multiple myeloma	115	1.103	1.12	1*	1.02 (0.64-1.62)	1.15 (0.71-1.85)	1.36 0.49-3.77)	1.52 (0.72-3.21)	0.89 (0.32-2.45)	1.20 (0.55-2.62)	0.86
All leukaemia	128	0.919	1.33	1*	1.22 (0.81-1.85)	0.85 (0.52-1.41)	0.63 (0.15-2.60)	1.42 (0.68-2.97)	0.20 (0.03-1.42)	0.74 (0.30-1.84)	0.43
All neoplasms	9487	112.09	90.96	1*	1.38 (1.31-1.45)	1.10(1.04-1.16)	1.07 (0.94-1.21)	1.19 (1.09-1.31)	1.41 (1.29-1.55)	1.63 (1.51-1.76)	<.0001

## Abbreviations

BMI: body mass index
CI: confidence interval
HR: hazard ratio
OR: odds ratio
RR: relative risk
SD: standard deviation
WHO: World Health Organization
